# Toll-like receptor 2 deficiency leads to delayed exacerbation of ischemic injury

**DOI:** 10.1186/1742-2094-9-191

**Published:** 2012-08-08

**Authors:** Ivan Bohacek, Pierre Cordeau, Mélanie Lalancette–Hébert, Dunja Gorup, Yuan-Cheng Weng, Srecko Gajovic, Jasna Kriz

**Affiliations:** 1 Laboratory for Neurogenetics and Developmental Genetics, Croatian Institute for Brain Research, School of Medicine, University of Zagreb, Salata 12, Zagreb, HR-10000, Croatia; 2 Centre de Recherche du Centre Hospitalier de l’Université Laval CHUL (CHUQ), 2705 boulevard Laurier, Québec, QC, G1V 4G2, Canada; 3 Department of Psychiatry and Neuroscience, Faculty of Medicine, Université Laval, Québec, Canada

**Keywords:** Apoptosis, IGF-1, microglia/macrophages, neuroinflammation, stroke, TLR2−/− mice

## Abstract

**Background:**

Using a live imaging approach, we have previously shown that microglia activation after stroke is characterized by marked and long-term induction of the Toll-like receptor (TLR) 2 biophotonic signals. However, the role of TLR2 (and potentially other TLRs) beyond the acute innate immune response and as early neuroprotection against ischemic injury is not well understood.

**Methods:**

TLR2−/− mice were subjected to transient middle cerebral artery occlusion followed by different reperfusion times. Analyses assessing microglial activation profile/innate immune response were performed using *in situ* hybridization, immunohistochemistry analysis, flow cytometry and inflammatory cytokine array. The effects of the TLR2 deficiency on the evolution of ischemic brain injury were analyzed using a cresyl violet staining of brain sections with appropriate lesion size estimation.

**Results:**

Here we report that TLR2 deficiency markedly affects post-stroke immune response resulting in delayed exacerbation of the ischemic injury. The temporal analysis of the microglia/macrophage activation profiles in TLR2−/− mice and age-matched controls revealed reduced microglia/macrophage activation after stroke, reduced capacity of resident microglia to proliferate as well as decreased levels of monocyte chemotactic protein-1 (MCP-1) and consequently lower levels of CD45^high^/CD11b^+^ expressing cells as shown by flow cytometry analysis. Importantly, although acute ischemic lesions (24 to 72 h) were smaller in TLR2−/− mice, the observed alterations in innate immune response were more pronounced at later time points (at day 7) after initial stroke, which finally resulted in delayed exacerbation of ischemic lesion leading to larger chronic infarctions as compared with wild-type mice. Moreover, our results revealed that TLR2 deficiency is associated with significant decrease in the levels of neurotrophic/anti-apoptotic factor Insulin-like growth factor-1 (IGF-1), expressed by microglia in the areas both in and around ischemic lesion.

**Conclusion:**

Our results clearly suggest that optimal and timely microglial activation/innate immune response is needed to limit neuronal damage after stroke.

## Background

Increasing evidence suggests that post-ischemic inflammation plays an important role in the evolution of brain injury after ischemia. However, to what extent inflammatory processes are deleterious and/or beneficial to brain recovery is a matter of debate and controversy [1-5]. One of the key features of the brain inflammatory response following ischemic injury is activation of the resident glial cells, including microglia. Activation of microglial cells after stroke is characterized by marked upregulation of the pattern-recognition receptors, such as Toll like receptors (TLRs) [6-14]. TLRs have the ability to bind two types of ligands: (i) pathogen-associated molecular pattern (PAMP) ligands in response to invading pathogens [15]; and (ii) endogenous danger-associated molecular pattern (DAMP) ligands, recognized in processes that present a threat to the structural integrity such as ischemic injury [12,16-19]. Recent evidence suggests that TLRs, especially TLR2, may play a key role in the evolution of brain damage following cerebral ischemia [9-11,20-23]. However, the exact role of TLR2 in modulation of the post-ischemic inflammatory response remains unclear. While previous studies were primarily focused on the role of TLR2 only in the acute phase 24 to 72 h after experimental stroke, the latter phases of the tissue response to ischemia remained unexplored [9,10,20,21,24]. Our recent work, using an *in vivo* imaging approach, revealed that the TLR2 response has a marked chronic component following brain ischemia [11]. Therefore, the aim of the work presented here was to investigate the role of TLR2 signaling and microglial activation beyond the 24 to 72 h time window.

Here, we report that TLR2 deficiency markedly affects neuroinflammatory response to ischemic brain injury resulting in altered post-ischemic inflammatory cell activation profiles, decreased proliferative capacity of resident microglia, reduced levels of MCP-1 and reduced accumulation of CD45^high^/CD11b^+^ cells at the site of the ischemic lesion. In addition, reduced levels of the neurotrophic and anti-apoptotic factor IGF-1 expressed by microglial cells were observed in TLR2-deficient mice. Importantly, cumulative effects of the altered post-ischemic inflammatory response resulted in a late increase of neuronal apoptosis and delayed exacerbation of ischemic lesion.

## Methods

### Experimental animals

All experiments were carried out on 2 to 4 month-old male TLR2−/− mice obtained from Jackson Laboratories (Bar Harbor, ME, USA), and wild-type (WT) C57BL/6 as a control group. Experimental procedures were approved by the Laval University Animal Care Ethics Committee and are in accordance with *The Guide to the Care and Use of Experimental Animals of the Canadian Council on Animal Care.*

### Surgical procedure

As previously described [3,11,25,26], unilateral transient focal cerebral ischemia was induced by intraluminal filament middle cerebral artery occlusion (MCAO) for 1 h followed by reperfusion periods of 1, 3, 4, 7 or 14 days. Briefly, all animals were anesthetized with 2% isoflurane, and body temperature was regularly checked and maintained at 37°C with a heating pad in order to avoid hypothermia. After midline neck incision, the left common carotid artery and ipsilateral internal and external carotid artery were exposed and isolated from surrounding tissue. A 12 mm long 6–0 silicon-coated monofilament suture (Doccol, CA, USA) was inserted via the proximal external carotid artery into the internal carotid artery and then into the circle of Willis, thus occluding the middle cerebral artery. After the occlusion period of 1 h the monofilament suture was removed, followed by different reperfusion periods. In our experiments we did not observe any increase in mortality in different experimental groups and, in general, mortality was very low. The whole operational procedure was conducted under the operating microscope. All animals were allowed *ad libitum* access to water and food before and after surgery.

### Tissue collection

After different reperfusion periods, all animals were anesthetized with an intraperitoneal injection of chloral hydrate (150 mg/kg, 300–350 μl solution, concentration 10 mg/ml), and transcardially perfused with 30 ml 0.9% saline, followed by ice-cold borax buffered 4% paraformaldehyde (PFA) at pH 9.5 (for *in situ* hybridization), or PBS buffered 4% PFA at pH 7.4 (for immunohistochemistry, immunofluorescent labeling and cresyl-violet staining). Tissue samples were then post-fixed overnight in 4% PFA and equilibrated in PBS buffered 30% sucrose for 48 h. Brains were embedded in Tissue-Tek (O.C.T. compound, Sakura, CA ,USA), frozen at −20°C, cut into 35 μm thick coronal sections using cryostat, and stored at −20°C.

In order to prepare samples for flow cytometric and cytokine antibody array analysis, animals were anesthetized with chloral hydrate and transcardially perfused with ice-cold 0.9% saline to remove all blood from the nervous tissue. Brain samples were surgically removed and immersed in Hibernate-A medium (for flow cytometric analysis), or immediately frozen in liquid nitrogen (for cytokine antibody array analysis).

### *In situ* hybridization

The expression and localization of TLR2 mRNA was detected using S^35^ labeled riboprobes. Protocols for probe synthesis and *in situ* hybridization were previously described by Lalancette-Hebert and colleagues [3,11].

### Immunofluorescence

Immunofluorescent labeling was performed on the brain sections of TLR2−/− mice and WT C57BL6 mice, collected 3 and 7 days after the MCAO procedure (all with visible stroke region, n = 4 animals/group/stage), according to the previously described procedure [3]. Briefly, brain sections were blocked for 30 minutes in PBS containing 10% goat serum and 0.25% Triton X-100. Sections were then incubated overnight at room temperature with primary antibody (1:250 mouse monoclonal anti-TLR2 (eBioscience, Burlington, Ontario, Canada), 1:500 rabbit polyclonal anti-Iba1 (Wako, Richmond, VA, USA), 1:2000 rat monoclonal anti-CD68 (AbD Serotec, Oxford, UK), 1:2000 rat polyclonal anti-BrdU (Axyll, Westbury, NY, USA), 1:400 mouse monoclonal anti-BrdU Alexa Fluor 488 conjugated (Molecular Probes, Eugene, OR, USA), 1:500 rat anti-Mac2 (American Type Culture Collection, Manassas, VA, USA), 1:400 rabbit polyclonal anti-cleaved caspase-3 (Cell Signaling, Danvers, MA, USA), 1:300 mouse monoclonal anti-NeuN (Millipore, Billerica, MA, USA), 1:1000 mouse monoclonal anti-glial fibrilary acidic protein (GFAP) (Millipore Billerica, MA, USA), 1: 50 rat monoclonal anti-CD11b (AbD Serotec) and 1:50 mouse monoclonal anti-IGF-1 (Millipore)). Afterwards, sections were incubated for 2 h at room temperature in corresponding secondary antibody 1:500 (Invitrogen, Eugene, OR, USA). Each of the steps above was followed by four 5 minute rinses in PBS-0.25% Triton X-100. At the end of the procedure, sections were coverslipped with Fluoromount G (Electron Microscopy Sciences, Fort Washington, PA, USA) and dried overnight.

### Immunohistochemistry

Immunohistochemistry was performed according to the previously described protocol [3,11]. Brain sections of TLR2−/− and WT mice were collected 3, 7 and 14 days after the MCAO procedure (all the stroke region, n = 4 brains/group). After an initial wash in PBS to remove O.C.T., endogenous peroxidase activity was attenuated with 0.6% hydrogen peroxide in PBS1X for 15 minutes. Afterwards, sections were blocked for 1 h in PBS1X containing 10% goat serum and 0.25% Triton X-100, and incubated overnight with 1:400 rabbit anti-cleaved caspase-3 antibody (Cell Signaling) in PBS1x containing 0.25% Triton X-100 and 5% goat serum. Sections were then incubated for 2 h with 1:500 goat anti-rabbit biotinylated secondary antibodies (Jackson ImmunoResearch, West Grove, PA, USA). For the amplification of the positive signal, incubation in avidin-biotin solution was performed (Vectastain ABC kit, Vector Laboratories, Burlingame, CA, USA). Staining was developed with nickel-DAB solution (0.3%) (Vector Laboratories). Each of the steps above was followed by four 5 minute rinses in PBS-0.25% Triton X-100. At the end of the procedure, sections were dehydrated and coverslipped with DPX mounting medium (Electron Microscopy Sciences).

### BrdU labeling

In order to visualize proliferating cells, mice were injected intraperitoneally with 5-bromo-2′-deoxyuridine (BrdU) (50 μg/g of mouse weight in saline) immediately before the MCAO procedure and again 2, 24 and 48 h after surgery. At 72 h after surgery, mice were anesthetized with an intraperitoneal injection of chloral hydrate (150 mg/kg, 300–350 μl solution, concentration 10 mg/ml) and transcardially perfused with 0.9% saline and PBS buffered 4% PFA. Brains were isolated, post-fixed overnight in 4% PFA, equilibrated in PBS buffered 30% sucrose for 48 h, and cut into 35 μm thick coronal sections. Afterwards, pretreatment was performed with 2.0 N HCl for protein hydrolysis (30 minutes, 37.0°C) with further neutralization of HCl with 0.1 M sodium borate (10 minutes), all diluted with KPBS. Immunofluorescent labeling was later performed on brain samples according to the standardized protocol described above.

### Flow cytometric analysis

In order to analyze the number of mononuclear cells and their proliferation properties after ischemia, flow cytometric analysis was performed on WT and TLR2−/− mice at 3 and 7 days after MCAO. As previously described [27,28], mice were injected intraperitoneally with BrdU 12, 24 and 36 h prior to sacrifice, according to BrdU Flow Kit protocol (BD, Franklin Lake, NJ, USA, USA). Collected brain samples were prepared for flow cytometric analysis according to previously described procedures [27,28]. Briefly, tissue was mechanically dissociated with a Teflon homogenizer and incubated with 1X HBSS containing papain (2 mg/ml) and DNase I (0.025 U/ml) for 30 minutes at 37°C. Afterwards, cell suspension was passed through 70 μm nylon cell strainer (BD). In order to isolate the mononuclear cell fraction, cell suspension was loaded in 30-37-70% isotonic Percoll gradient (GE Healthcare, Uppsala, Sweden) and centrifuged for 40 minutes at 500 g. After centrifugation, an interphase containing mononuclear cells was collected, washed thoroughly, and stained with anti-CD45 conjugated with V500 (BD Horizon), and anti-CD11b conjugated with allophycocyanin (BD). Further, for proliferation analysis, cells were stained with anti-BrdU conjugated with fluorescin isothiocyanate antibody, following BrdU Flow Kit procedure (BD). Samples were afterwards analyzed on a flow cytometer (BD LSRII) immediately after the end of the staining protocol by a ‘blinded’ individual. Cells were gated using front scatter and side scatter to eliminate unviable cells. Further gating was performed using side scatter and CD45 in order to select the inflammatory cell population. Afterwards, two-color analysis with the use of anti-CD11b and anti-CD45 antibodies was performed in order to delineate CD45^low^/CD11b^+^ (that is, microglial) and CD45^high^/CD11b^+^ (that is, macrophage-like) cell populations [27,28]. Proliferation analysis was performed using two-color analysis of anti-CD11b and anti-BrdU antibodies. Non-stroked, BrdU-treated animals were used as negative controls for proliferation analysis.

### Cytokine array

The expression profile of inflammatory cytokines was performed with a Mouse Cytokine Antibody Array (Raybio Mouse Inflammation Antibody Array 1 (Cat#AAM-INF-1), RayBiotech, Norcross, GA, USA). As previously described [3,29] , protein samples were obtained by homogenization of TLR2−/− and WT brains 1 and 4 days after MCAO (n = 3/group) in 1x cell lysis buffer that was included in the RayBiotech kit (Raybio®Mouse Inflammation Antibody Array 1.1, Ray Biotech, #AAM-INF-1 L). Protein lysates were obtained by homogenization of brain of control and transgenic mice in 1X Cell Lysis Buffer (included in the Ray Biotech kit) with Protease inhibitor cocktail (Sigma #P8340, St. Louis, MO, USA). After extraction, samples were spun down and supernatant was used for the experiment. The protein concentration was determined for each sample and diluted at 300 μg total protein in 1x blocking buffer. For each group (three mice per group) samples were pooled together and incubated with the array membrane overnight at 4°C. After washing in the washing buffer (included in the RayBiotech kit), membranes were incubated with biotin-conjugated antibodies overnight. After washing, membranes were incubated with horseradish peroxidase conjugated streptavidin diluted in the blocking buffer for 2 h. Signal detection was performed according to RayBiotech protocol, by exposing membranes to x-ray film (Biomax MR1; #8701302; Kodak, Rochester, NY, USA), and the obtained results analyzed with ImageJ software.

### Assessment of the infarct area

Every sixth coronal cryostat section of TLR2−/− and WT mouse brains collected after reperfusion periods of 3, 7 and 14 days after MCAO (n = 10/group) was stained with cresyl-violet according to the standardized protocol, coverslipped with DPX mounting medium (Electron Microscopy Sciences) and afterwards digitized. The area of infarction (‘direct stroke area’) was quantified with the ImageJ program (version 1.42q for Windows, National Institutes of Health, USA), and the infarct volume was calculated and expressed in mm^3^. As previously described [10], correction for edema was applied for brains reperfused for 3 days by calculating the brain swelling. First, the ‘indirect stroke area’ was calculated as contralateral (non-stroked) hemisphere area minus non-stroked area of the ipsilateral (infarcted) hemisphere. A difference between ‘direct’ and ‘indirect’ stroke area represented brain swelling. The correction of ‘direct stroke area’ was performed by subtracting brain swelling volume from ‘direct stroke area’. In addition, ‘indirect stroke area’ represents the volume of healthy tissue that correlates to the volume of ischemic tissue not affected by post-ischemic shrinkage. Thus, ‘direct stroke area’ reflects lesion consolidation (shrinkage) and glial scar formation and ‘indirect stroke area’ represents lesion progression (that is, how does altered inflammatory response reflect on surrounding-cell survival and lesion dynamics).

### Quantification and statistical analysis

#### *Immunofluorescence*

For the quantification of the immunofluorescent signal (Iba1, CD68, BrdU, Mac2, IGF-1), four fields of view per section (every sixth section, all stroke area), ten sections per animal were acquired on a fluorescent microscope (Leica DM5000B, Solms, Germany) using a 20x Plan Fluotar objective. Immunoreactivity was quantified with the ImageJ software by measuring the integrated optical density (intensity of fluorescence per unit of surface area), and results were expressed in arbitrary units as previously described [3]. The data were averaged and analyzed by a two-tailed unpaired Student’s *t*-test.

#### *Immunohistochemistry*

Every sixth sample section through the brain was used for the immunohistochemical labeling with anti-cleaved caspase-3 antibody. The number of positively stained cells was estimated by the optical fractionator method using a Nikon Eclipse 80i microscope equipped with Stereo Investigator software. The ipsilateral (ischemic) hemisphere was traced using a 4 Plan Apochromat and sampled using 60 Plan Apochromat objectives (Nikon, El Segundo, CA, USA). The counting parameters for the experiments were the counting frame size (width and height 100 × 100 μm), sampling grid (500 × 500 μm), dissector height (10 μm) and the guard-zone thickness (1.5 μm). Counts were performed on the entire ischemic area of the ipsilateral (ischemic) hemisphere. Results were expressed as number of cells per mm^3^ of ischemic tissue. The data were averaged and statistically analyzed by a two-tailed unpaired Student’s *t*-test.

#### *Cytokine array*

Cytokine expression assays were quantified by measuring the optical density of each cytokine spot on the membrane with ImageJ software, as previously described [3,29]. Cytokines were presented on membranes as duplicates and the analysis was performed twice. The background values were subtracted from cytokine expression values. Data were expressed in arbitrary units relative to appropriate positive control. Further, data were averaged and analyzed by a two-tailed unpaired Student’s *t*-test.

## Results

### Microglial cells upregulate Toll-like receptor 2 in response to transient ischemic brain injury

**Figure 1:**
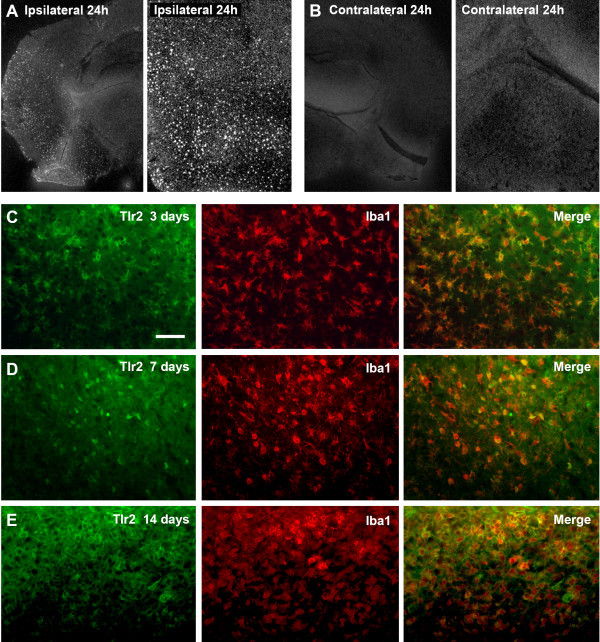
**Microglial cells upregulate Toll-like receptor 2 in response to transient ischemic brain injury. (A, B)** Photomicrographs of *in situ* hybridization for Toll-like receptor (TLR) 2 mRNA in wild type (WT) mice 24 h after ischemic injury, indicating marked induction of TLR2 on the ipsilateral side shown in low (A, left) and high (A, right) magnification, in contrast to the contralateral side where no positive S^35^ signal was observed (B). **(C, D, E)** Double labeling 3, 7 and 14 days after transient middle cerebral artery occlusion (MCAO) reveals co-localization of TLR2 (green) and Iba1 (red) in WT mice, indicating that TLR2 are expressed almost exclusively by the microglia/macrophage cell population in both the early and late phase of the response to ischemic brain injury. Scale bar: C, 50 μm.

To identify whether TLR2 is indeed upregulated in microglial cells (at mRNA and protein levels) after stroke we performed a series of *in situ* hybridization experiments followed by double immunofluorescence analysis. In accordance with previous studies [9,11,20], 24 h after stroke we observed a marked increase of the TLR2 mRNA in the area of ischemic lesion and the peri-infarct zone, showing a positive S^35^*in situ* hybridization signal (Figure 1A). In contrast, no signal was observed in the contralateral, unlesioned hemisphere (Figure 1B). As shown in Figure 1C,D,E, double immunofluorescence analysis of the brain sections after MCAO revealed a co-localization of TLR2 staining with Iba1 (a common marker of microglial cells). The co-localization was observed in activated microglial cells situated in ischemic and peri-infarct regions of the brain at 3, 7 and 14 days post-stroke, suggesting that microglia/macrophages upregulate TLR2 in early, as well as in more chronic phases, of the brain response to ischemic injury (Figure 1C,D). As previously reported [11], few neurons found within the ischemic core area were positive for TLR2, while we did not observe any co-localization between TLR2 and GFAP-positive astrocytes (data not shown).

### Toll-like receptor 2 deficiency alters microglia/macrophage activation profiles

**Figure 2:**
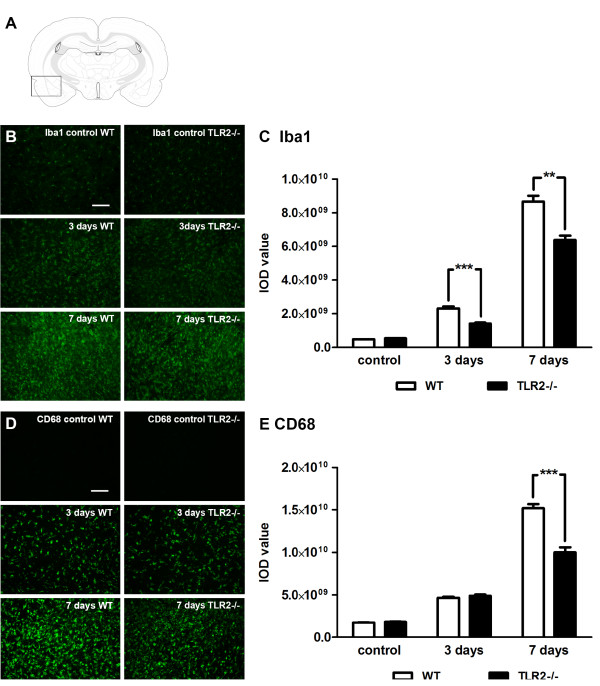
**Toll-like receptor 2 deficiency leads to altered microglia/macrophage activation profiles. (A)** Schematic representation of the brain section used for the immunohistochemical experiments. Box represents the area magnified in B and D, and in Figure 3A,C,F,G, as well as in Figures 6 and 7. **(B)** Representative photomicrographs of Iba1 immunoreactivity in control (unlesioned) wild-type (WT) and Toll-like receptor (TLR) 2−/− mice, as well as 3 and 7 days after transient middle cerebral artery occlusion (MCAO), indicate reduced accumulation of Iba1-positive cells in TLR2 deficient mice. **(C)** Quantification of Iba1 immunoreactivity revealed significantly decreased immunoreactivity in TLR2−/− mice compared with the WT group of mice 3 and 7 days after transient MCAO. **(D)** Photomicrographs of CD68 immunoreactivity in unlesioned (control) WT and TLR2−/− mice 3 and 7 days after transient ischemic lesion. **(E)** Quantitative analysis of CD68 immunoreactivity in both groups reveals significant reduction of the signal intensity 7 days after MCAO in the TLR2−/− group. Values (C, E) indicate mean ± SEM (n = 4; **P <0.001 ***P <0.0001). Scale bars: B, D: 100 μm.

Because activated microglial activation in response to ischemic injury is characterized by a robust upregulation of TLR2, we next investigated how and whether this process may be affected in the context of TLR2 deficiency. First, we analyzed expression levels of the distinct microglia/macrophage activation markers such as Iba1 and CD68. The analyses were performed 3 and 7 days following transient MCAO. As expected, immunofluorescent labeling indicated a significant increase of Iba1 immunoreactivity in the ischemic side of the brain. The relative increase of Iba1 immunoreactivity (as compared with unlesioned brain controls) was also observed in TLR2-deficient mice. However, comparison of the levels of microglial activation after stroke revealed a marked reduction of Iba1 immunoreactivity in the brain section of TLR2−/− mice as compared with WT mice, at both 3 and 7 days after MCAO (Figure 2B). Quantitative analysis of Iba1 staining revealed significant reduction at 3 days (1.63-fold) as well as 7 days (1.36-fold) after MCAO in TLR2−/− mice when compared with WT mice (Figure 2C) (control: WT, 0.48 × 10^9^ ± 0.16 × 10^9^, n = 4; TLR2−/−, 0.54 × 10^9^ ± 0.06 × 10^9^, n = 4, *P* = 0.438; 3 days: WT, 2.31 × 10^9^ ± 0.12 × 10^9^ , n = 4; TLR2−/−, 1.41 × 10^9^ ± 0.06 × 10^9^, n = 4, *P* <0.001; 7 days: WT, 8.67 × 10^9^ ± 0.34 × 10^9^, n = 4; TLR2−/−, 6.37 × 10^9^ ± 0.26 × 10^9^, n = 4, *P* = 0.002). Similar results were obtained following analysis of CD68 immunoreactivity. As shown in Figure 2D,E quantitative analysis of CD68 immunoreactivity revealed no significant difference at the lesion site between WT and TLR2−/− mice 3 days after transient MCAO. Interestingly, however, CD68 immunoreactivity was significantly decreased in TLR2−/− mice 7 days after MCAO, indicating a 1.52-fold reduction in signal intensity (Figure 2E) (control: WT, 1.73 × 10^9^ ± 0.36 × 10^9^, n = 4; TLR2−/−, 1.83 × 10^9^ ± 0.48 × 10^9^, n = 4, *P* = 0.258; 3 days: WT, 4.65 × 10^9^ ± 0.13 × 10^9^; TLR2−/−, 4.90 × 10^9^ ± 0.15 × 10^9^, n = 4, *P* = 0.265; 7 days: WT, 15.19 × 10^9^ ± 0.47 × 10^9^, n = 4, TLR2−/−, 9.99 × 10^9^ ± 0.60 × 10^9^, n = 4, *P* <0.001).

### Decreased levels of monocyte chemotactic protein-1 (MCP-1) and CD45^high^/CD11b^+^ cells in ischemic brains of Toll-like receptor 2−/− mice

**Figure 3:**
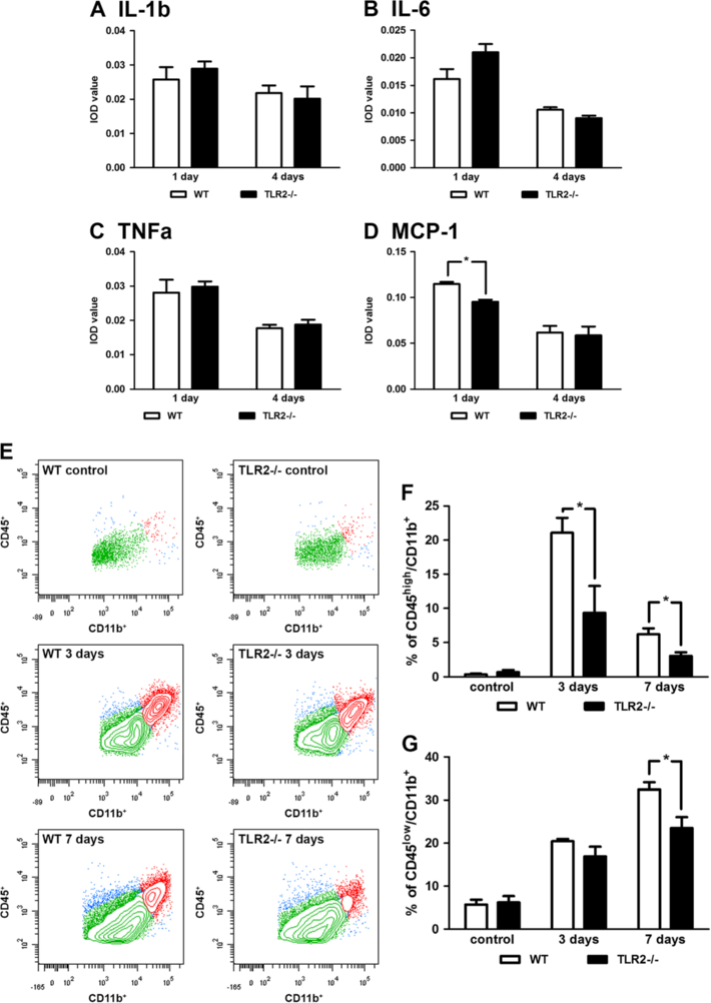
**Decreased levels of MCP-1 and CD45**^**high**^**/CD11b**^**+**^**expressing cells in Toll-like receptor 2 deficient mice. (A-C)** Expression analysis of inflammatory cytokines IL-1β, IL-6 and TNF-α on protein levels 1 and 4 days after transient middle cerebral artery occlusion (MCAO) revealed no significant difference between wild-type (WT) and Toll-like receptor (TLR) 2−/− groups. **(D)** Significant reduction of MCP-1 levels in the TLR2−/− group compared with their WT controls 1 day after transient MCAO. **(E)** Topographic representation of isolated brain mononuclear cells from ischemic hemispheres of control, WT and TLR2−/− mice 3 and 7 days after stroke, analyzed using two-color flow cytometry. Cells were analyzed for CD45 and CD11b, thus allowing us to distinguish two different cell populations: CD45^high^/CD11b^+^ (that is, the macrophage-like) population (red); and CD45^low^/CD11b^+^ (microglial) population (green). **(F)** Flow cytometric analysis showed a significant decrease in the number of CD45^high^/CD11b^+^ cells in TLR2−/− mice compared with WT mice at both 3 and 7 days after stroke. **(G)** Quantitative analysis of CD45^low^/CD11b^+^ cells (microglia) indicates a significant reduction in cell numbers in TLR2−/− mice as compared with WT 7 days after stroke, while 3 days after stroke the difference is not significant, but there is a tendency toward reduced cell numbers in TLR2−/− mice. Values (A-D) are expressed as mean ± SEM (n = 4, *P <0.05). Data (F, G) are expressed as percentage of CD45^+^ events ± SEM (n = 4; *P <0.05).

**Figure 4:**
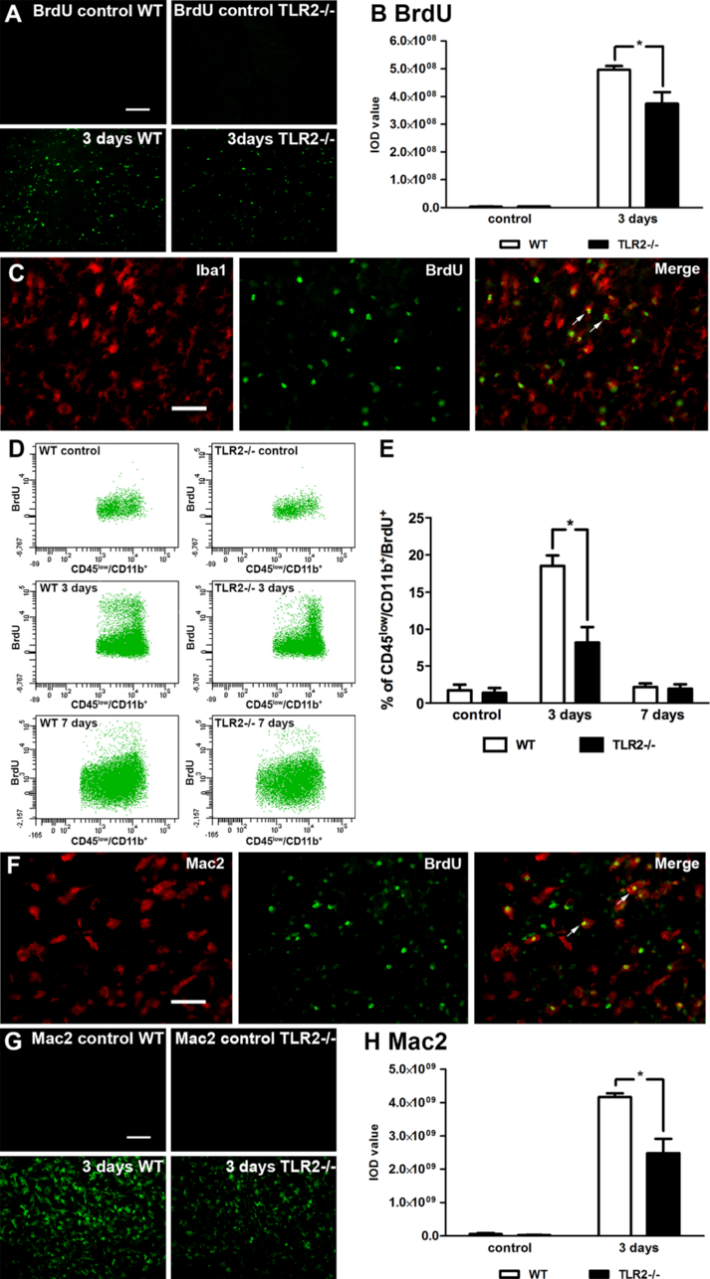
**Toll-like receptor 2 deficient mice display reduced proliferation of resident microglial cells. (A)** Photomicrographs of BrdU immunoreactivity in wild-type (WT) and Toll-like receptor (TLR) 2−/− mice 3 days after transient middle cerebral artery occlusion (MCAO) and their unlesioned controls, respectively. **(B)** Quantification of BrdU immunoreactivity indicates reduced cell proliferative capacity in TLR2−/− mice compared with WT mice 3 days after transient MCAO. **(C)** Representative photomicrographs of double immunostaining on WT brain sections 3 days after transient MCAO show almost complete co-localization of BrdU (green) positive cells with Iba1 (red; white arrows). **(D)** Flow cytometric analysis of the CD45^low^/CD11b^+^ (microglial) population proliferation in WT and TLR2−/− mice 3 days after MCAO compared with their contralateral (unlesioned) hemisphere controls. **(E)** Graph presents quantification of microglial proliferation measured by flow cytometric analysis. Data indicate reduced microglial proliferation in TLR2−/− mice compared with WT mice 3 days after MCAO. **(F)** Double immunofluorescent labeling reveals co-localization of BrdU (green) and Mac-2 (red; white arrows) in WT brain sections 3 days after transient MCAO. **(G)** Representative photomicrographs of Mac-2 immunofluorescence in WT and TLR2−/− mice observed 3 days after transient MCAO. **(H)** Quantification of Mac-2 immunoreactivity 3 days after transient MCAO revealed reduced signals in TLR2−/− mice compared with WT mice. Data (B, H) are indicated as mean ± SEM (n = 4; *P <0.05). Data (E) are expressed as percentage of CD45^+^ events ± SEM (n = 4; *P <0.05). Scale bars: A, G, 100 μm; C, F, 50 μm.

Evidence suggests that pro-inflammatory cytokines such as IL-1β, IL-6 and TNFα may influence the extent of neuronal damage after cerebral ischemia [1,5]. To determine whether deficient microglial activation in TLR2−/− mice after stroke (as shown in the results above) induced changes in profiles of pro-inflammatory cytokines, we used a standard mouse cytokine antibody array technique to measure over 40 different cytokines from ischemic brains of WT and TLR2−/− mice [3,29]. Because production of pro-inflammatory cytokines peaks within 24 h following ischemic/reperfusion injury (with the exception of transforming growth factor β levels at 4 days), time points of 1 and 4 days post-stroke were chosen for the analysis [30]. Ischemic injury was associated with a robust increase in the production of pro-inflammatory cytokines both in WT and TLR2−/− mice. To our surprise, quantitative analysis revealed no significant changes in the levels of pro-inflammatory cytokines such as IL-1β, IL-6 and TNFα between the two experimental groups (Figure 3A,B,C) (IL-1β at 1 day: WT, 0.026 ± 0.004, n = 4; TLR2−/− 0.029 ± 0.002, n = 4, *P* = 0.602; at 4 days: WT, 0.218 ± 0.002, n = 4; TLR2−/− 0.202 ± 0.004, n = 4, *P* = 0.700; IL-6 at 1 day: WT, 0.016 ± 0.002, n = 4; TLR2−/− 0.021 ± 0.001, n = 4, *P* = 0.082; at 4 days: WT, 0.011 ± 0.001, n = 4; TLR2−/− 0.009 ± 0.001, n = 4, *P* = 0.058; TNFα at 1 day: WT, 0.028 ± 0.003, n = 4; TLR2−/− 0.030 ± 0.001, n = 4, *P* = 0.720; at 4 days: WT, 0.018 ± 0.001, n = 4; TLR2−/− 0.019 ± 0.001, n = 4, *P* = 0.535). Interestingly, however, we observed a significant decrease in the levels of MCP-1 1 day after MCAO (Figure 4D) in TLR2−/− mice compared with their WT controls (1 day: WT, 0.115 ± 0.002, n = 4; TLR2−/− 0.095 ± 0.002, n = 4, *P* = 0.026; 4 days: WT, 0.062 ± 0.007, n = 4; TLR2−/− 0.059 ± 0.009, n = 4, *P* = 0.841). Because it has been largely acknowledged that MCP-1 plays a pivotal role in monocyte recruitment to the site of injury, we next investigated whether recruitment of infiltrating cells may have been affected in the context of TLR2 deficiency. One of the established protocols for evaluation of the numbers of infiltrating monocytes and resident microglial cells is based on the two-color quantitative and qualitative flow cytometric analysis of CD45^+^/CD11b^+^ cell populations [27,28]. Figure 3E presents a topographic representation of the number of CD45^high^/CD11b^+^ cells (that is, macrophage-like; red) and CD45^low^/CD11b^+^ cells (that is, microglia; green) in the control (unlesioned) hemisphere and in the ischemic hemisphere 3 and 7 days after stroke in WT and TLR2−/− mice. As shown in Figure 3F, quantitative analysis revealed a 2.26-fold reduction in numbers of CD45^high^/CD11b^+^ cells in ischemic brains of TLR2−/− mice 3 days after MCAO and a 2.02-fold reduction 7 days after MCAO compared with WT mice (control: WT: 0.35 ± 0.12%, n = 4; TLR2−/−, 0.70 ± 0.30%, n = 4, *P* = 0.325; 3 days: WT, 21.08 ± 2.19%; TLR2−/−, 9.34 ± 3.94%, n = 4, *P* = 0.040; 7 days: WT, 6.20 ± 0.86%; TLR2−/−, 3.06 ± 0.54%, n = 4, *P* = 0.210). We next analyzed numbers of the resident microglial cells after stroke. The quantitative flow cytometric analysis indicated a significant 1.38-fold reduction in numbers of the CD45^low^/CD11b^+^ cells in stroked brains of TLR2−/− mice as compared with WT 7 days after stroke (Figure 3G), suggesting a decrease in population of the resident microglial cells. At 3 days after stroke the difference is not statistically significant, but a tendency towards a reduced cell number (1.21-fold reduction) can be observed in TLR2−/− mice compared with WT group (control: WT: 5.71 ± 1.15%, n = 4; TLR2−/−, 6.23 ± 1.47%, n = 4, *P* = 0.793; 3 days: WT, 20.44 ± 0.55%; TLR2−/−, 16.91 ± 2.26%, n = 4, *P* = 0.137; 7 days: WT, 32.44 ± 1.69%; TLR2−/−, 23.55 ± 2.52%, n = 4, *P* = 0.026). Our results suggest that early and selective downregulation of MCP-1 together with a significant decrease in the numbers of CD45^high^/CD11b^+^ and CD45^low^/CD11b^+^ cells suggest that TLR2 may have a role in the modulation of monocyte/brain macrophage recruitment to the site of ischemic injury.

### Reduced proliferation of resident microglial cells in ischemic brains of Toll-like receptor 2 deficient mice

A highly characteristic feature of the injury-induced microglial activation is massive, but usually transient, expansion of microglial cell population, peaking 48 to 72 h after initial insult [3,31]. Our results suggest a decrease in resident microglia numbers. However, at present, it is unclear to what extent the ischemic injury-induced microglial proliferation is affected in the context of TLR2 deficiency. To address this issue, the TLR2−/− and WT mice were injected daily with BrdU. The BrdU-positive/proliferating cells were quantified 3 and 7 days after stroke. In agreement with previous results, quantitative analysis of the BrdU staining (Figure 4A,B) revealed a robust increase in BrdU immunoreactivity in WT mice. As revealed in Figure 4B, we observed a 24.55% reduction in cell proliferation in TLR2−/− mice (control: WT, 0.03 × 10^8^ ± 0.01 × 10^8^, n = 4; TLR2−/−, 0.04 × 10^8^ ± 0.01 × 10^8^, n = 4, *P* = 0.278; 3 days: WT, 4,97 × 10^8^ ± 0,13 × 10^8^; TLR2−/−, 3,75 × 10^8^ ± 0,42 × 10^8^, n = 4, *P* = 0.048). To further confirm that BrdU is indeed expressed in proliferating microglial cells, double immunofluorescence for BrdU and Iba1 (markers of cell proliferation and microglia/macrophages, respectively) 3 days after transient MCAO was performed. As expected, results demonstrated that the majority of BrdU-positive cells were also positive for Iba1 staining (Figure 4C). To increase the resolution of the results obtained by BrdU signal intensity measurement the microglial proliferative capacity in TLR2−/− mice was assessed by flow cytometry measurement. Proliferation analysis was carried out on the population of CD45^low^/CD11b^+^ cells (microglial population) [27,28] obtained from TLR2−/− and WT animals injected with BrdU (Figure 4D). The flow cytometric analysis 3 days after stroke revealed a robust, 10-fold increase in the number of CD45^low^/CD11b^+^/BrDU^+^ cells in WT mice (Figure 4D,E). As further shown in Figure 4E, quantitative analysis revealed a significant (55.91%) reduction in the number of proliferating CD45^low^/CD11b^+^ cells in TLR2−/− compared with WT mice (control (contralateral): WT, 1.72 ± 0.78%, n = 4; TLR2−/−, 1.39 ± 0.63%, n = 4, *P* = 0.762; 3 days: WT, 18.51 ± 1.41%; TLR2−/−, 8.16 ± 2.14%, n = 4, *P* = 0.014). The number of proliferating cells declined by day 7 and there were no significant differences in the numbers of CD45^low^/CD11b^+^/BrDU^+^ cells between the two experimental groups (Figure 4E).

Previously we have shown that the injury-activated resident microglial cells in proliferation upregulate galectin-3 (a Mac-2 marker of activated microglia) [3]. Further characterization of the proliferating cells in our experimental model revealed that approximately 80% of BrdU positive cells co-localize with Mac-2 (Figure 4F). Interestingly, analysis of the Mac-2 immunoreactivity revealed a significant 40.52% reduction in the signal in TLR2−/− compared with WT mice (Figure 4G,H) (control (contralateral): WT, 0.06 × 10^9^ ± 0.03 × 10^9^, n = 4; TLR2−/−, 0.03 × 10^9^ ± 0.01 × 10^9^, n = 4, *P* = 0.448; 3 days: WT, 4,17 × 10^9^ ± 0,20 × 10^9^; TLR2−/−, 2,48 × 10^9^ ± 0,75 × 10^9^, n = 4, *P* = 0.019). Altogether, the obtained results indicate that TLR2 deficiency may affect ischemic injury-induced proliferation of the resident microglial cells.

### Toll-like receptor 2 deficiency increases delayed neuronal apoptosis and exacerbates ischemic injury

**Figure 5:**
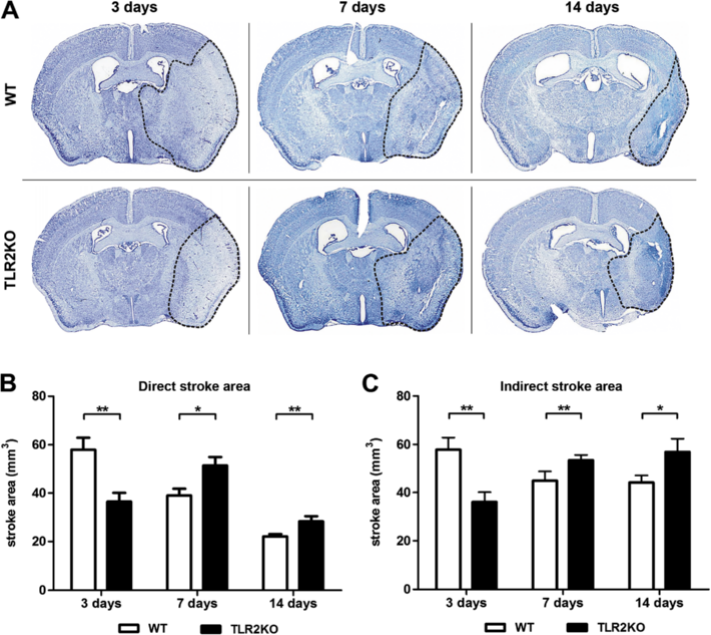
**Toll-like receptor 2 deficiency delays evolution of ischemic brain lesion. (A)** Representative low-magnification photomicrographs of cresyl violet stained brain sections of wild type (WT) and Toll-like receptor (TLR) 2−/− mice 3, 7 and 14 days after transient middle cerebral artery occlusion (MCAO). The ischemic area is emphasized by a black dashed line. **(B)** Significantly reduced direct stroke area in TLR2−/− mice 3 days after transient MCAO compared with WT mice. This was followed by marked lesion exacerbation in TLR2−/− mice 7 days after injury, while lesions in WT mice showed a reduction in size. The evolution of direct stroke area stagnated 14 days after injury, indicating significantly larger final lesion size in TLR2−/− mice compared with the WT group. **(C)** A smaller indirect stroke area in TLR2−/− mice compared with WT mice 3 days after transient MCAO. In contrast, 7 days after injury, indirect stroke area in the TLR2−/− group was larger compared with WT mice, and the same relationship was observed 14 days after ischemic injury. Values are expressed as mean ± SEM (n = 10, *P <0.05, **P <0.001).

We have demonstrated that activated/proliferating and galectin-3-positive microglial cells exert neuroprotective properties by secreting IGF-1 [3]. To date, it is unclear whether defective microglial activation/proliferation after stroke may affect evolution of the ischemic injury in TLR2-deficient mice. Although previous studies have reported smaller acute ischemic lesions in TLR2-deficient mice and/or in mice treated with TLR2 blocking antibodies, the analysis has been limited to the first 24 to 72 h after stroke [9,10,20,21]. We next asked whether initial neuroprotection against ischemic injury in TLR2−/− mice is also extended to later time points after stroke. Quantification of the ischemic lesions was performed using cresyl violet stained brain sections of TLR2−/− mice and their WT controls (n = 10/group) 3, 7 and 14 days after MCAO. As expected, TLR2−/− mice showed significantly reduced direct stroke area (37.03%) compared with WT mice 3 days after MCAO (Figure 5A5B), which is in accordance with previous reports [20,21]. As described in detail in the Methods section and previously reported [10], we analyzed “indirect stroke area” as a measure of contralateral (non-stroked) hemisphere area minus non-stroked area of the ipsilateral (infarcted) hemisphere. The difference between ‘direct’ and ‘indirect’ stroke area represented brain swelling. The correction of ‘direct stroke area’ was then performed by subtracting brain swelling volume from ‘direct stroke area’. In addition, ‘indirect stroke area’ represents the volume of healthy tissue that correlates to the volume of ischemic tissue not affected by post-ischemic shrinkage. Thus, ‘direct stroke area’ reflects lesion consolidation (shrinkage) and glial scar formation, while ‘indirect stroke area’ represents lesion progression (that is, the effect of altered inflammatory response on lesion dynamics). In that way, ‘direct’ and ‘indirect’ stroke area represent important parameters for adequate characterization of more chronic time points.

To our surprise, 7 days after transient MCAO, the size of direct stroke area in TLR2−/− mice increased and was significantly larger (31.87%) when compared with controls. Due to a post-ischemic consolidation of the ischemic brain tissue and glial scar formation in the later phase (14 days after transient MCAO), direct stroke area of WT and TLR2−/− mice were reduced compared with 3 and 7 days after MCAO. Still, direct stroke area remained significantly larger in TLR2−/− mice when compared with the WT control group (27.99%) (3 days: WT, 57.92 ± 4.98, n = 10; TLR2−/−, 36.47 ± 3.58, n = 10, *P* = 0.003; 7 days: WT, 39.00 ± 2.78, n = 10; TLR2−/− 51.43 ± 3.47, n = 10, *P* = 0.012; 14 days: WT, 22.15 ± 0.96, n = 9; TLR2−/− 28.35 ± 2.09, n = 6, *P* = 0.010). Quantitative assessment of indirect stroke area (volume of healthy tissue that correlates to volume of damaged, ischemic tissue not affected by post-ischemic consolidation and glial scar formation) 3 days after transient MCAO revealed a significant reduction of indirect stroke area (37.58%) in TLR2−/− mice compared with the control WT group (Figure 5A,C). In addition, 7 days after transient MCAO, indirect stroke area was significantly larger (26.41%) in the group of TLR2−/− mice compared with controls. In the later phase (14 days after transient MCAO), evolution of the ischemic lesion stagnated, and indirect stroke area in TLR2−/− mice remained significantly larger (29.07%) compared with the WT control group (3 days: WT, 57.82 ± 5.04, n = 10; TLR2−/−, 36.09 ± 4.04, n = 10, *P* = 0.004; 7 days: WT, 42.29 ± 3.24, n = 10; TLR2−/− 53.46 ± 2.19, n = 10, *P* = 0.010; 14 days: WT, 44.10 ± 3.08, n = 9; TLR2−/− 56.92 ± 5.43, n = 6, *P* = 0.049). Altogether, these results indicate that TLR2 deficiency induces delayed exacerbation in the size of the ischemic lesion.

**Figure 6:**
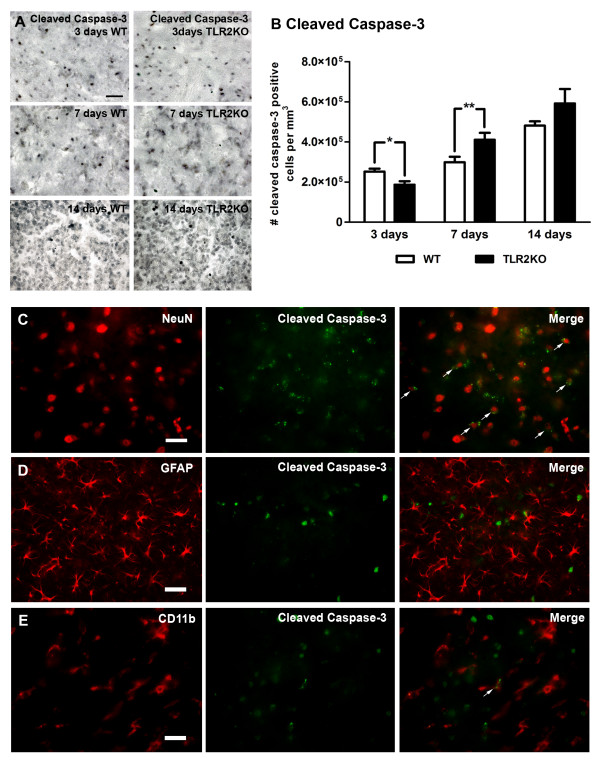
**Delayed exacerbation of neuronal apoptosis in Toll-like receptor 2 deficient mice. (A)** Photomicrographs of cleaved caspase-3 immunoreactivity in wild-type (WT) and Toll-like receptor (TLR) 2 −/− mice 3,7 and 14 days after transient middle cerebral artery occlusion (MCAO). **(B)** Quantification of labeled cells showed a higher number of apoptotic cells in WT compared with TLR2−/− mice 3 days after injury, while 7 days after injury the number of cleaved caspase-3-positive cells was significantly higher in the TLR2−/− group compared with the WT group of mice. The number of cleaved caspase-3-positive cells was higher in TLR2−/− mice 14 days after transient MCAO, but not significantly because of high variation within the group. **(C)** Double immunofluorescence analysis reveals a high proportion of cleaved caspase-3 (green) positive cells correlating with the neuronal marker NeuN (red, co-localization marked with white arrows). **(D)** No co-localization was observed for GFAP (red) and cleaved caspase-3 (green). **(E)** Only a few cleaved caspase-3-positive cells (green) showed co-localization with Iba1 (red, co-localization marked with white arrow heads), while the vast majority were not co-localizing. Data (B) are expressed as mean ± SEM (n = 4, *P <0.05, **P <0.001). Scale bars: A, C, D, E, 25 μm.

Next, we investigated whether the late increase in the size of ischemic lesions detected in TLR2−/− mice is associated with an increase in delayed neuronal cell death. The number of apoptotic cells in both groups was determined using immunohistochemical labeling with anti-cleaved caspase-3 antibody 3, 7 and 14 days after transient MCAO. As shown in Figure 6A,B, the number of apoptotic cells 3 days after transient MCAO showed a 1.35-fold reduction in TLR2−/− mice compared with the control WT group, suggesting an early neuroprotective effect of TLR2 deficiency. However, consistent with a delayed increase in the size of ischemic lesions, at 7 days after transient MCAO the number of cleaved caspase-3-positive cells per mm^3^ of infarcted volume in TLR2−/− mice showed a 1.37-fold increase compared with the group of WT mice. At 14 days after transient MCAO, the number of cleaved caspase-3-positive cells remained higher in the TLR2−/− group compared with the WT group, although not significantly higher due to a large variation in the number of apoptotic cells in the TLR2−/− group (3 days: WT, 2.52 × 10^5^ ± 0.15 × 10^5^, n = 4; TLR2−/−, 1.87 × 10^5^ ± 0.18 × 10^5^, n = 4, *P* = 0.046; 7 days: WT, 2.99 × 10^5^ ± 0.27 × 10^5^, n = 4; TLR2−/−, 4.11 × 10^5^ ± 0.35 × 10^5^, n = 4, *P* = 0.046; 14 days: WT, 4.81 × 10^5^ ± 0.21 × 10^5^, n = 4; TLR2−/−, 5.92 × 10^5^ ± 0.72 × 10^5^, n = 4, *P* = 0.2128). In order to investigate which cell types underwent apoptosis, double immunofluorescence labeling of cleaved caspase-3 and the neuronal marker NeuN, the astrocyte marker GFAP, and the microglia/macrophage marker CD11b was performed on brain sections of WT mice 3 days after transient MCAO (Figure 6C,D,E). The cleaved caspase-3 signal co-localized to a great degree (>90%) with the neuronal marker NeuN (Figure 6C). As revealed in Figure 6E, only a few caspase-3-positive cells were also positive for the microglia/macrophage marker CD11b. We did not observe co-localization of cleaved caspase-3 immunostaining with the astrocyte marker GFAP (Figure 6D). Hence, delayed exacerbation of ischemic injury in TLR2−/− mice is characterized by a delayed increase in neuronal apoptosis.

### Reduced levels of Insulin-like growth factor 1 in brains of Toll-like receptor 2 deficient mice after transient ischemia

**Figure 7:**
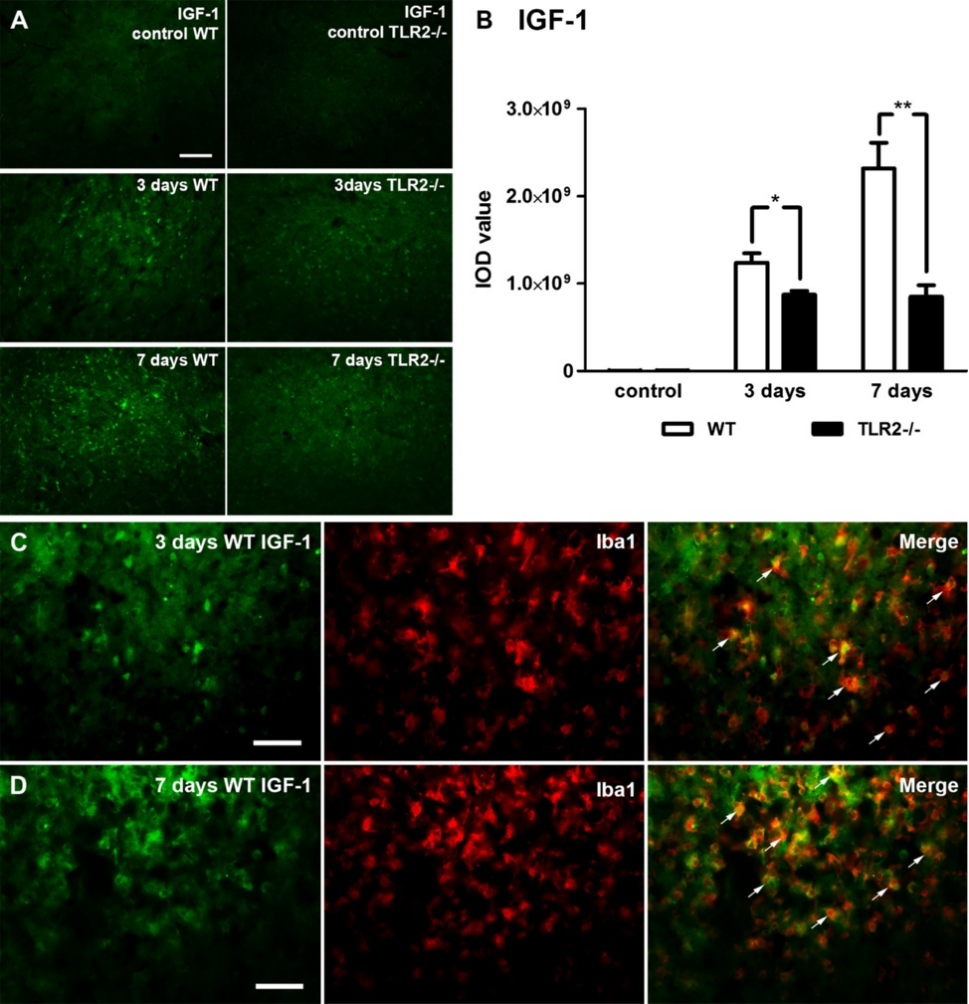
**Reduced levels of Insulin-like growth factor 1 in brains of Toll-like receptor 2 deficient mice after stroke. (A)** Photomicrographs of **(**IGF)-1 immunoreactivity in control (unlesioned) and lesioned brain hemispheres at 3 and 7 days after stroke, showing reduced levels of IGF-1 immunoreactivity in Toll-like receptor (TLR) 2−/− mice. **(B)** Quantification of signal intensity indicates a reduced level of IGF-1 present in the brains of TLR2−/− mice following transient ischemia compared with wild-type (WT) mice. **(C, D)** Double immunofluorescence labeling reveals that IGF-1 expression was present in microglia/macrophages within and around the infarcted region in both 3 and 7 days after ischemia. Data (B) are indicated as mean ± SEM (n = 4; *P <0.05, **P <0.001). Scale bars: A, 100 μm; C, D: 50 μm.

Previous findings demonstrated that activated and proliferating microglial cells produce neurotrophic/anti-apoptotic factors such as IGF-1 and thus exert neuroprotection [3,32,33]. Because our results demonstrated reduced proliferating capacity/decrease in numbers of microglial cells in TLR2−/− mice, we hypothesized that IGF-1 levels may have been affected in TLR2 deficiency which may have resulted in an increase in delayed neuronal death. To test our hypothesis, we measured IGF-1 levels in control (unlesioned) and lesioned hemispheres of WT and TLR2−/− mice (Figure 7A,B). Analyses were performed 3 and 7 days following transient ischemia. As expected, immunofluorescence labeling indicated a significant increase of signal intensity between control (unlesioned) and lesioned hemispheres in WT and TLR2−/− mice. Interestingly, quantitative analysis of the IGF-1 signal revealed a significant 1.42-fold reduction at 3 days and a 2.73-fold reduction at 7 days after MCAO in TLR2−/− mice when compared with the WT mice group (Figure 7B) (control (contralateral): WT, 0.004 × 10^9^ ± 0.001 × 10^9^, n = 4; TLR2−/−, 0.006 × 10^9^ ± 0.001 × 10^9^, n = 4, *P* = 0.283; 3 days: WT, 1.24 × 10^9^ ± 0.11 × 10^9^; TLR2−/−, 0.87 × 10^9^ ± 0.04 × 10^9^, n = 4, *P* = 0.039; 7 days: WT, 2.32 × 10^9^ ± 0.29 × 10^9^, n = 4, TLR2−/−, 0.85 × 10^9^ ± 0.13 × 10^9^, n = 4, *P* = 0.004). To further confirm that the cells expressing IGF-1 after transient ischemia are microglia, double labeling with the marker Iba1 was performed 3 and 7 days after stroke. IGF-1 expression on the ischemic site was almost exclusively due to microglia/macrophages (Figure 7C,D), which is in agreement with previous studies [3,33]. The same results were obtained on stroked tissues of TLR2−/− mice (data not shown). No cell-specific immunostaining was detected for astrocytes or neurons in WT or TLR2−/− at both time points on the ischemic lesion site (data not shown). Altogether, these results suggest that the defective immune response in the brain caused by TLR2 deficiency resulted in a reduced amount of microglia-secreted IGF-1 (a very potent anti-apoptotic molecule for stressed neurons) in and around the ischemic lesion site which may have caused an increase in delayed neuronal death and consequent exacerbation of the ischemic injury observed in TLR2-deficient mice.

## Discussion

Microglial activation and innate immune response are key features of the brain inflammatory response to ischemic injury. Using a live imaging approach we have previously shown that microglial activation after stroke is characterized by a marked long-term induction of the TLR2 signals, thus suggesting an important role of TLR2 signaling in brain ischemia. In the present study, we report altered microglia activation profiles and delayed exacerbation of ischemic injury in the mouse model lacking functional TLR2 receptors. Namely, the TLR2 deficiency resulted in: (1) reduced microglia/macrophage activation after stroke; (2) reduced capacity of resident microglia to proliferate; and (3) decreased levels of MCP-1 and consequently lower levels of CD45^high^/CD11b^+^ expressing cells. Importantly, although acute ischemic lesions (24 to 72 h) were smaller in TLR2−/− mice, the observed alterations in the innate immune response were more pronounced at later time points (at day 7) after initial stroke, which finally resulted in delayed exacerbation of the ischemic lesion leading to larger chronic infarctions as compared with WT mice. Finally, reduced microglial activation/proliferation resulted in a significant decrease in expression of IGF-1 in microglial cells after stroke. Altogether, our results clearly suggest that optimal and timely microglial activation/innate immune response is needed to limit the extent of neuronal damage after stroke.

Upon activation, microglial cells start to express TLRs on their surface [11,34,35]. The functional role of TLRs is the signal transduction into the cell via TLR domain-containing adaptor proteins and MyD88 adaptor protein resulting in degradation of IκB proteins and translocation of transcription factor NFκB into the nucleus, which induces expression of proinflammatory cytokines in response to PAMPs and DAMPs [5,36,37]. Among diverse TLRs, it has been reported that, after transient cerebral ischemia, induction of TLR2 predominates among TLRs, followed by TLR4 and TLR9 [9]. After brain injury and/or pathogen exposition, TLR2 is mainly expressed in the population of microglial cells [11,20,38,39]. In addition, some reports indicate expression of TLR2 in astrocytes, oligodendrocytes and ependymal cells, as well as neurons and neuronal progenitor cells [12,18,40,41]. Our results revealed a marked induction of TLR2 (at the mRNA and protein level) that was restricted to ischemic lesion and peri-infarct zones. No signal was observed contralaterally, that is on the non-stroke side. Further, our analysis revealed that, in early and late phases of the brain response to transient ischemia, upregulation of TLR2 occurred almost exclusively on activated microglial cells (see Figure 1C,D,E). Minor expression was found in a limited subpopulation of neurons situated in the ischemic core area and we did not observe any co-localization between TLR2 and GFAP-stained astrocytes. These results are in agreement with previous reports on TLR2 induction following brain injury from ours [11] and other laboratories [9,20,21,23,40,42].

As discussed, previous studies reported initial neuroprotection against ischemia in TLR2−/− mice [9,20,21]. In keeping with previous work, the results of our study revealed and confirmed that, in TLR2−/− mice, the early post-stroke period (up to 3 days after transient MCAO) is characterized by smaller infarcts (see Figure 5). Interestingly, however, initial neuroprotection was not extended to later time points and we observed progressive and delayed exacerbation of neuronal damage in TLR2-deficient mice. Thus, in TLR2-deficient mice an early reduction in microglial response was associated with initially smaller lesions; a long-term decrease in microglia/macrophage activation and proliferation led to increased neuronal apoptosis and delayed exacerbation of ischemic lesion (Figures 2,3,4,5 and 6). Previous work suggests that some inflammatory molecules may have time-dependent functions [43]; however, the role of TLR2 (and potentially other TLRs) beyond the acute innate immune response in ischemic injury is not well understood. The results presented in this study suggest that TLR2 has a role in injury-induced monocyte/macrophage recruitment as well as in colony proliferation. Increased activation and accumulation of the myeloid cells on the site of the lesion after stroke may occur either due to massive proliferation of resident microglia, which normally peaks 48 to 72 h after initial activation, or due to increased monocyte recruitment from the circulation to the lesion site [3,31,44,45]. Our data suggest that both processes may have been affected in TLR2−/− mice. We detected a selective decrease in MCP-1 levels 1 day after stroke and consequently lower numbers of CD45^high^/CD11b^+^ expressing cells at both 3 and 7 days after the onset of ischemia. In addition, TLR2 deficiency was associated with the reduced capacity of resident microglial cells to proliferate. Reduced microglial proliferation was observed in stroked brains of TLR2−/− mice 3 days after MCAO. Although at present we cannot exclude the possibility that the detected decrease in microglial proliferation may be due to initially smaller infarcts, it is important to mention that the size of infarction did increase over time in TLR2−/− mice; however, this was not accompanied by an increase in microglial proliferative response and/or activation. As shown by flow cytometric analysis, the proliferative response stayed low in TLR2−/− mice 7 days after stroke (see Figure 4). On the other hand, a decrease in CD45^high^/CD11b^+^ expressing cells observed at days 3 and 7, and a selective decrease in MCP-1 observed in TLR2−/− mice, suggest that TLR2 signaling is needed for adequate recruitment of circulating monocytes. Taken together, these results support in part the hypothesis established on the axonal injury model which suggests that, in the acute phase, TLR2 may modulate proliferation rather than the entry of circulating monocytes [23,31,46-48].

The question that arises here is how to correlate deficient monocyte/microglia responses observed in TLR2−/− mice with delayed exacerbation of the ischemic injury. One of the possibilities is that, as previously reported, rapid phagocytic removal of dead or dying cells prevents the release of proinflammatory intracellular components and contributes to the resolution of inflammation [49]. Reduced CD68 signal intensity may suggest a reduced number of phagosomes present on the lesion site in TLR2−/− mice, thus implying that the engulfment of cellular debris is altered with a consequently prolonged inflammation. It is noteworthy that altered microglia/macrophage activation profiles observed in TLR2−/− mice were more pronounced at day 7 post-stroke, which coincided with the initial enlargement of ischemic lesions in TLR2-deficient mice. An additional explanation as suggested by our results is that a defective innate immune response in the brain caused by TLR2 deficiency results in deficient microglia proliferation and consequently reduced levels of IGF-1, which may lead to an increase in delayed neuronal death and exacerbation of ischemic injury.

Based on our results, we may conclude that reduction in numbers as well as aberrant profiles of the activated microglia/macrophages may over time result in a microenvironment that is detrimental for neuronal survival and causes exacerbation of ischemic lesions.

## Conclusion

Taken together, our data suggest that functional TLR2 signaling is required for modulation of microglia/macrophage responses in the injured brain. Moreover, our data also indicate that TLR2 induction may exert temporally differential effects. However, the ischemic injury-induced TLR2 response in the brain is instrumental for timely resolution of the post-ischemic inflammation, finally limiting exacerbation and/or propagation of the initial ischemia-induced neuronal damage.

## Abbreviations

DAMP, danger associated molecular pattern; GFAP, glial fibrilary acidic protein; IGF, Insulin-like growth factor −1; IL, interleukin; MCAO, middle cerebral artery occlusion; MCP, Monocyte chemotactic protein-1; PAMP, pathogen-associated molecular pattern; PBS, phosphate-buffered saline; PFA, paraformaldehyde; TLR, Toll like receptor; TNF, tumor necrosis factor; WT, wild-type.

## Competing interests

The authors declare that they have no competing interests.

## Authors’ contributions

IB carried out the immunoassays, cytokine array, flow cytometry analysis, quantifications and wrote the manuscript. PC participated in cytokine array, flow cytometry and participated in study design. MLH performed *in situ* hybridization and participated in study design. DG participated in study design and performed the statistical analysis. YCW performed surgical procedures and helped in tissue collection and cryo-cutting. SG participated in study design and coordination. JK conceived the study, participated in study design and coordination and wrote the manuscript. All authors read and approved the final manuscript.
